# Downregulation of epithelial sodium channel (ENaC) activity in cystic fibrosis cells by epigenetic targeting

**DOI:** 10.1007/s00018-022-04190-9

**Published:** 2022-04-25

**Authors:** Giovanna Blaconà, Roberto Raso, Stefano Castellani, Silvia Pierandrei, Paola Del Porto, Giampiero Ferraguti, Fiorentina Ascenzioni, Massimo Conese, Marco Lucarelli

**Affiliations:** 1 Department of Experimental Medicine, Sapienza University of Rome, Rome, Italy; 2 Department of Biomedical Sciences and Human Oncology, University of Bari, Bari, Italy; 3 Department of Biology and Biotechnology “Charles Darwin”, Sapienza University of Rome, Rome, Italy; 4 Department of Medical and Surgical Sciences, University of Foggia, Foggia, Italy; 5 Pasteur Institute, Cenci Bolognetti Foundation, Sapienza University of Rome, Rome, Italy

**Keywords:** Cystic fibrosis, Epithelial sodium channel (ENaC), CFTR, Epigenetic therapy

## Abstract

The pathogenic mechanism of cystic fibrosis (CF) includes the functional interaction of the cystic fibrosis transmembrane conductance regulator (CFTR) protein with the epithelial sodium channel (ENaC). The reduction of ENaC activity may constitute a therapeutic option for CF. This hypothesis was evaluated using drugs that target the protease-dependent activation of the ENaC channel and the transcriptional activity of its coding genes. To this aim we used: camostat, a protease inhibitor; S-adenosyl methionine (SAM), showed to induce DNA hypermethylation; curcumin, known to produce chromatin condensation. SAM and camostat are drugs already clinically used in other pathologies, while curcumin is a common dietary compound. The experimental systems used were CF and non-CF immortalized human bronchial epithelial cell lines as well as human bronchial primary epithelial cells. ENaC activity and *SCNN1A*, *SCNN1B* and *SCNN1G* gene expression were analyzed, in addition to *SCNN1B* promoter methylation. In both immortalized and primary cells, the inhibition of extracellular peptidases and the epigenetic manipulations reduced ENaC activity. Notably, the reduction in primary cells was much more effective. The *SCNN1B* appeared to be the best target to reduce ENaC activity, in respect to *SCNN1A* and *SCNN1G*. Indeed, SAM treatment resulted to be effective in inducing hypermethylation of *SCNN1B* gene promoter and in lowering its expression. Importantly, CFTR expression was unaffected, or even upregulated, after treatments. These results open the possibility of CF patients’ treatment by epigenetic targeting.

**Supplementary Information:**

The online version contains supplementary material available at 10.1007/s00018-022-04190-9.

## Introduction

The epithelial Na^+^ channel (ENaC) has a pivotal role in regulating salt and fluid transport at cellular apical side in epithelia of many organs including the lung [1, 2]. The ENaC has the functional properties of a Na^+^ channel with high Na^+^ selectivity, low conductance and amiloride sensitivity. It is expressed in human epithelial cells that line the distal renal tubule, distal colon, several exocrine glands and the upper and lower airways. It is also expressed in lung epithelial progenitors and stem cells with a crucial role in development and regeneration of the respiratory epithelium. ENaC is a heterotrimeric channel composed of 3 transmembrane subunits, the *α*, *β* and *γ* coded by 3 genes with sequence similarities: *SCNN1A *[3], *SCNN1B* and *SCNN1G *[4]. Genetic diseases are caused by either loss-of-function (for example, pseudohypoaldosteronism type I, PHA-I) [5] or gain-of-function (for example, Liddle’s syndrome [6]) variants in ENaC genes. Additionally, SNPs of *SCNN1A* gene are associated with neonatal respiratory distress syndrome [7].

ENaC activation occurs by the proteolytic cleavage of *α* and *γ* subunits leading to an increase in channel conductance [8, 9]. Different classes of proteases act to activate ENaC, including intracellular proteases such as furin, extracellular proteases such as prostasin and the soluble/secreted proteases trypsin and neutrophilic elastase. Other mechanisms contributing to regulate ENaC activity include intracellular second messengers, such as the cAMP/PKA complex, and protein interactions with the cystic fibrosis transmembrane conductance regulator (CFTR) channel and with the extracellular short-palate lung and nasal epithelial clone 1 (SPLUNC1) [10]. While interaction with CFTR mainly affects ENaC open probability (Po) [11], SPLUNC1 limits ENaC activity by reducing its density on the apical membrane of the epithelial cells [12, 13].

Cystic fibrosis (CF) is a monogenic disease caused by pathogenic variants of the *CFTR* gene, encoding a cAMP/PKA-activated chloride channel, with multifaceted phenotypical manifestations [14, 15]. CF shows a complex relationship between genotype and phenotype originated from several sources of variability, often not taken into account during genetic studies [16, 17]. These sources of variability can be more directly related to CFTR [18, 19], or may depend on the dense network of direct and indirect interactions of CFTR with other cellular proteins [20, 21]. In effect, CFTR and ENaC regulate the hydration of human airways by a physiologic dual ion transport [22–24]. CFTR and ENaC drive, respectively, fluid secretion and absorption with a complex and not completely understood interplay. CFTR exerts an inhibitory effect on ENaC by a combination of a decreased average open probability and a reduced channel expression at the cell surface [25, 26]. Importantly, by interacting with ENaC, wild-type CFTR impedes proteolysis and suppresses channel opening, whereas mutated CFTR fails to protect ENaC from proteolytic cleavage and activation [26]. CFTR/ENaC cross-talk disruption, possibly based on ENaC deregulation and/or molecular lesions, has been proposed to contribute to CF lung disease and/or to CFTR-related disorders (CFTR-RD) [27–30]. A role of ENaC as a concomitant pathogenic factor when only one or no copy of CFTR is mutated, or as modifier of phenotype [31] when both copies of the CFTR are mutated, is now recognized. The deregulation of ENaC, rather than its variants, seems more frequently involved, often through modification at transcriptional or protein level [8, 23, 26, 32, 33].

The functional interaction between CFTR and ENaC highlighted the targeting of ENaC as a potential therapeutic strategy for CF [34, 35]. The ENaC over-expression hypothesis, confirmed by the CF-like lung disease of the *β*-ENaC overexpressing transgenic mice [36], suggested that ENaC inhibition could ameliorate the unbalanced ion transport through CF epithelia. The attenuation of ENaC activity has been attempted by a variety of approaches, such as amiloride [37, 38], RNA interference [39–42] and modulators [43, 44]. However, after the failure of the first generation of pharmacological inhibitors, a change of mindset is mandatory, also to choose between the use of ENaC targeting as monotherapy or in parallel to other treatments focused on CFTR [10, 45–48].

The structure of ENaC genes suggests a role for DNA methylation. The *SCNN1G* and *SCNN1B* genes have, respectively, two [49, 50] and one [51] CpG islands. The *SCNN1A* gene has a high density of CpG sites, although not organized in a CpG island [52]. In effect, DNA methylation can induce transcription changes of the *SCNN1G* gene [50] and of *SCNN1B* gene, in the last case also correlated to gastric cancer [53, 54] and essential hypertension [55]. We have recently shown that the expression modulation of ENaC genes depends in part on the DNA methylation patterns of specific DNA regions [56]. The experimental evidences about a DNA methylation-dependent transcription of ENaC genes, point to epigenetics and chromatin remodeling as new therapeutic opportunities for CF [50, 57], although barely explored till now. In addition, the role of non-CpG methylation has not been studied in ENaC genes so far, although it has been demonstrated to be crucial in other contexts [58–62].

In this work we investigated the possibility to modulate ENaC transcription and activity by epigenetic modulators and protease inhibitors, using immortalized and primary CF human bronchial epithelial cell lines. We evaluated the effect of DNA hypermethylation, chromatin condensation and protease inhibition on the coordinated action of CFTR and ENaC, measured as airway surface fluid reabsorption ability. Epigenetic manipulations was carried out using curcumin [63] (a polyphenolic compound in turmeric with many roles as epigenetic modulator) and S-adenosyl methionine [64] (involved in DNA methylation of cytosines) with the final goal to lower ENaC activity. In particular, we have explored the effects of these drugs on the DNA methylation pattern, both CpG and non-CpG, of the *SCNN1B* gene, which in turn affects its transcriptional level.

## Materials and methods

### Cells and culture conditions

Two immortalized human bronchial epithelial cell lines were used: 16HBE14o- (16HBE) cells expressing wild-type CFTR and CFBE41o- (CFBE) cells expressing the most common mutated CFTR genotype (F508del/F508del). 16HBE and CFBE cells, kindly provided by Dieter Gruenert, were grown as reported [65]. Briefly, cells were cultured in DMEM (Sigma-Aldrich, Saint Louis, Missouri, USA) supplemented with 10% FBS, 1% L-glutamine and 1% Penicillin/Streptomycin (all from Euroclone, Milan, Italy) in fribonectin-collagen coated plastics.

Non-CF and CF-derived (F508del/F508del) human bronchial primary epithelial cells were also used (called, respectively, primary HBE and primary CF HBE). Primary cells were provided by the Italian Cystic Fibrosis Research Foundation (FFC) Facility (Molecular Genetics Laboratory, Gaslini Institute, Genoa, Italy) and cultured according to the supplier’s specifications. Four samples of primary cells, from two different CF patients and two different wild-type individuals were used at passage two. Briefly, cells were first expanded in LHC9/RPMI 1640 (1:1) serum-free medium (Euroclone) and then seeded on porous supports (Snapwell, Corning Costar) at 5 × 10^5^ cells/cm^2^. Finally, cells were induced to differentiate at the “air–liquid interface” (ALI) in LHC9/Ham’s F12 (1:1, v:v) supplemented with 2% Ultroser G serum substitute (provided by the FFC facility), 2 mM L-glutammine, 100 U/mL penicillin and 100 μg/mL streptomycin (all from Euroclone). Supports were coated with rat-tail collagen diluted 1/100. All cell lines were cultured at 37 °C under 5% CO_2_.

### Biochemical treatments

To investigate gene expression, methylation and fluid absorption, different concentrations of drugs were tested. Cells were treated for 24 h before the analysis with camostat mesylate (Sigma-Aldrich) at concentrations ranging from 0.3 µM to 30 µM [44]. Amiloride (100 µM and 10 μM), dexamethasone (50 nM), S-adenosyl methionine (100 µM) and curcumin (6 µM) (Sigma-Aldrich), were also used [66] (concentrations selected also after dosage testing, data not shown).

### Trans-epithelial electrical resistance (TEER)

To assess differentiation in primary cultures, measurement of TEER was performed using chopstick electrodes coupled with an ohmmeter (Millicell-ERS, Millipore, Billerica, Massachusetts, USA), after the addition of 500 µL medium to the apical surface of cultures. Epithelial differentiation was achieved for TEER values of at least 400–500 Ω/cm^2^.

### Apical fluid absorption

Trans-epithelial fluid transport measurements were performed according to a previously reported protocol [66]. Briefly, the apical surface of epithelial cells was washed three times using 200 µL of saline solution containing 137 mM NaCl, 2.7 mM KCl, 8.1 mM Na_2_HPO_4_, 1.5 mM KH_2_PO_4_, 1 mM CaCl_2_ and 0.5 mM MgCl_2_ (all from Sigma-Aldrich). Next, the apical surface of cultures was incubated with 50 μL of the same saline solution (with or without drugs) overlaid with 150 μL of mineral oil to prevent evaporation. After 24 h, the apical fluid was recovered and then centrifuged to separate the oily phase. This allowed to measure the residual volume of aqueous phase. Fluid absorption is reported as μL/(cm^2^ × h).

### DNA/RNA extraction and quantification

Total RNA was extracted from 1.5 × 10^6^ immortalized cells or 1 × 10^6^ primary cells using Trizol Reagent (Invitrogen, Carlsbad, CA, USA). The quality of the extracted RNA was evaluated by the identification of 28S, 18S and 5S RNA bands in agarose 1% gel.

Genomic DNA was extracted using QIAamp DNA Mini Kit (Qiagen, Manchester, U.K.).

Quantification and purity of both RNA and DNA were evaluated using a spectrophotometer (Nanodrop, ThermoFisher Scientific, Waltham, MA, USA).

### Real-time PCR and expression analysis

DNase treatment was performed to remove residual amounts of contaminating genomic DNA according to the following protocol: RNA (2 μg) was incubated with 0.4 units of DNase I (New England Biolabs, Ipswich, MA, USA) at 37 °C for 10 min. Subsequently, the sample was treated with 1X EDTA (5 mM, pH = 8.0, Sigma-Aldrich) at 75°C for 10 min, to deactivate the enzyme.

After DNase treatment, 2 μg RNA were reverse transcribed using Reverse Trascription System kit (Promega, Fitchburg, WI, USA), containing both oligo-dT and random primers, according to the manufacturer’s protocol.

Real-time PCR was performed using SYBR Green PCR Master Mix (Applied Biosystems, Forster City, CA, USA) according to the supplier’s specification in an ABI7500 Real Time PCR system (Applied Biosystems). Real-time PCR primers are reported in Table 1. Once acquired the threshold cycles (C_T_) of the individual genes, the analysis was performed using ΔC_T_ calculated as the difference between the C_T_ values of the target genes of interest (*SCNN1A*, *SCNN1B*, *SCNN1G* and *CFTR*) and the average C_T_ of the housekeeping gene (*β-actin)*. The ΔΔC_T_ was calculated as the difference between the ΔC_T_ of the gene in the experiment and the ΔC_T_ of the gene in the control. Fold-change was calculated as 2^(−ΔΔCT) ^and indicated as relative quantification (RQ). Average values were calculated using the results of three independent experiments.

**Table 1:** Primers used for real-time PCR

Name	Sequence 5ʹ-3ʹ	T_a_	Amplicon
*β*-actin forward	5ʹ-GCCGGGACCTGACTGACTA-3ʹ	62 °C	204 bp
*β*-actin reverse	5ʹ-TGGTGATGACCTGGCCGT-3’		
SCNN1A (*α*-ENaC) forward	5ʹ-GCTGATAACCAGGACAAAACACAA-3ʹ	60 °C	68 bp
SCNN1A (*α*-ENaC) reverse	5ʹ-CGTCGCTGGGCAGGAA-3ʹ		
SCNN1B (*β*-ENaC) forward	5ʹ-GAGCCCTGCAACTACCGGA-3ʹ	60 °C	101 bp
SCNN1B (*β*-ENaC) reverse	5ʹ-GCCGAAGGAAGTGCCTTCTC-3ʹ		
SCNN1G (*γ*-ENaC) forward	5ʹ-GCCCTGAAGTCCCTGTATGG-3ʹ	60 °C	101 bp
SCNN1G (*γ*-ENaC) reverse	5ʹ-CGGTGGGAGAATCTAGGCTG-3ʹ		
CFTR forward	5ʹ-AAGCGTCATCAAAGCATGCC-3ʹ	60 °C	110 bp
CFTR reverse	5ʹ-TTGCTCGTTGACCTCCACTCA-3ʹ		

### DNA methylation analysis and bisulfite sequencing

**Fig. 1:**
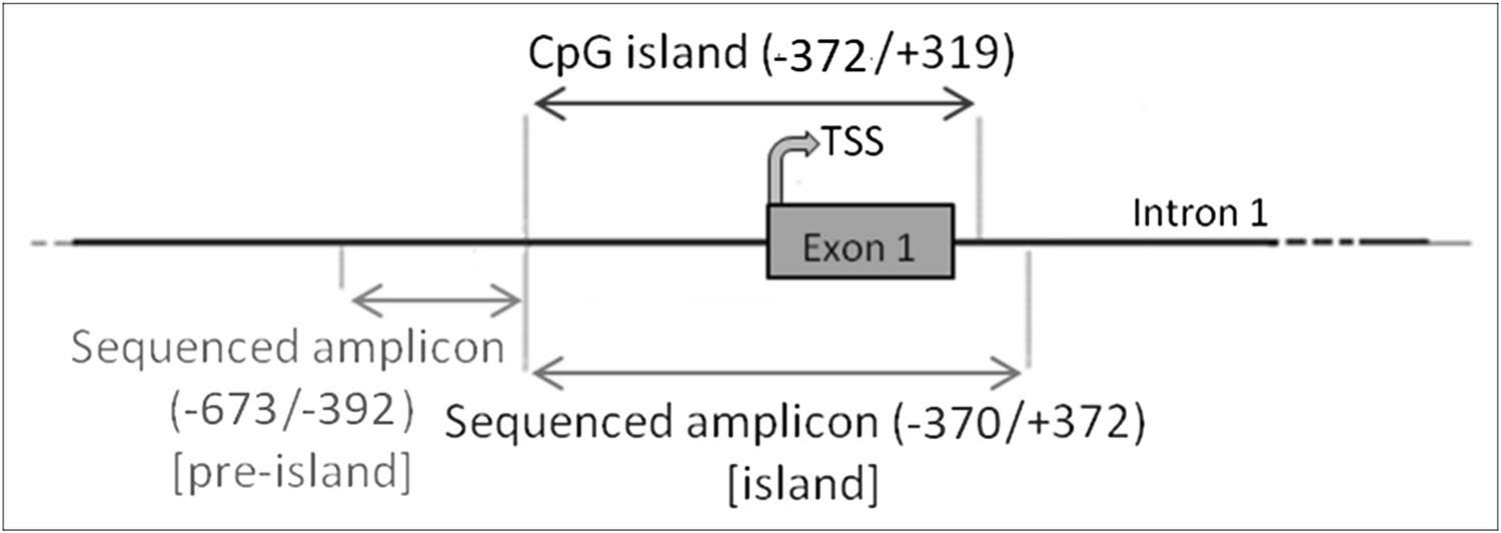
Genomic regions of SCNN1B investigated for methylation analysis in CFBE and CF primary bronchial epithelial cells. The first zone investigated, the pre-island, covers the region from -673 to -392, contains 56 cytosines and is located upstream to the CpG island. The second zone studied, spans the CpG island, covers the region from − 370 to + 372, contains 236 cytosines and includes the exon 1 and the TSS

DNA methylation studies were performed in both CFBE and CF-derived primary cells. The entire region analyzed consists of 1013 base pairs (86 base pairs including primers were excluded) of the 5ʹ-flanking region of *SCNN1B* (*β*-ENaC) gene sequence (ENSG00000168447), from position − 673 to position + 372. The region of interest was divided in two zones: a first zone upstream the CpG island (named “pre-island”) ranging from position − 673 to position − 392 and a second zone spanning the CpG island (named “island”) ranging from position − 370 to position + 372 (Fig. 1).

Bisulfite treatment of 700 ng genomic DNA was performed by EpiTect Bisulfite kit (Qiagen). Bisulfite DNA conversion exploits the different sensitivities of cytosine and 5-methylcytosine to undergo deamination by bisulfite, under acidic conditions [67]. The treatment results in the conversion of cytosine to uracil, whereas the 5-methylcytosine remains non-reactive. This conversion creates non-complementary strands that are amplified by PCR. Pairs of primers insensitive to cytosine conversion were synthesized with degenerated bases avoiding an underestimation of DNA methylation, in particular of non-CpG methylation [68, 69]. The primer sequences are reported in Table 2.

**Table 2:** Primers used for DNA methylation analysis

Region	Forward	Reverse	T_a_
Pre-island	5ʹ-GTGG^C^/_T_TGAAATGATAGT^C^/_T_^C^/_T_TGAAA^C^/_T_^C^/_T_TT-3ʹ	5ʹ-CACCCCT^G^/_A_CA^G^/_A_ACACA^G^/_A_T^G^/_A_TCCTCT^G^/_A_-3ʹ	58 °C
Island	5ʹ-^C^/_T_TTG^C^/_T_^C^/_T_^C^/_T_AGAGGA^C^/_T_A^C^/_T_TGTGT^C^/_T_TG^C^/_T_AGG-3ʹ	5ʹ-TCT^G^/_A_CACCCT^G^/_A_^G^/_A_^G^/_A_^G^/_A_^G^/_A_CTTTTCCCA^G^/_A_C-3ʹ	58 °C

Next, PCR products were inserted into pCR2.1 plasmid using TA Cloning kit, and then introduced in the One Shot TOP10 Chemically Competent *E. coli* (all from Invitrogen) through bacterial transformation, according to the manufacturer’s instructions. Positive colonies were screened by the addition of 40 μg X-gal (Invitrogen) in LB agar medium (Sigma-Aldrich) and subsequent colony PCR.

Al least 30 clones for each replicate samples were sequenced and analyzed using the ABI PRISM 3130*xl* Genetic Analyzer (Applied Biosystems); modified cytosines were recognized by comparison with the reference DNA sequence. DNA methylation analysis was performed using BiQ Analyzer software v 2.0 (Max Planck Institut Informatik).

### Statistical analysis

For fluid absorption, real-time PCR and TEER assays, data were represented as mean ± standard deviation (SD), of at least 3 independent experiments. Statistical difference between control and drug-treated samples was evaluated using ANOVA and Bonferroni’s post test.

The methylation of a specific C moiety represents the ratio between the number of clones where that moiety is methylated and the number of all the analyzed clones (for that moiety), expressed as average percentage of at least 3 independent experiments ± SD. The overall methylation is a measurement, expressed as average percentage ± SD, of the total methylation level of a specific group of C (overall C; CpG; non-CpG; CCTCC), calculated as the number of methylated C of that group divided by the total number of analyzed C (including also all other C not belonging to that group). For the evaluation of the pattern at single C level, statistical difference between control and drug-treated samples was evaluated using contingency tables and *χ*^2^ test. Whenever absolute frequencies were below the value of 5, the results of adjacent cytosines were cumulated to make the contingency table analysis applicable. For the evaluation of average methylation levels, ANOVA and Bonferroni’s post test were applied.

For all analyses, a *P *< 0.05 was considered statistically significant.

## Results

### Fluid absorption in CFBE and 16HBE cells

**Fig. 2:**
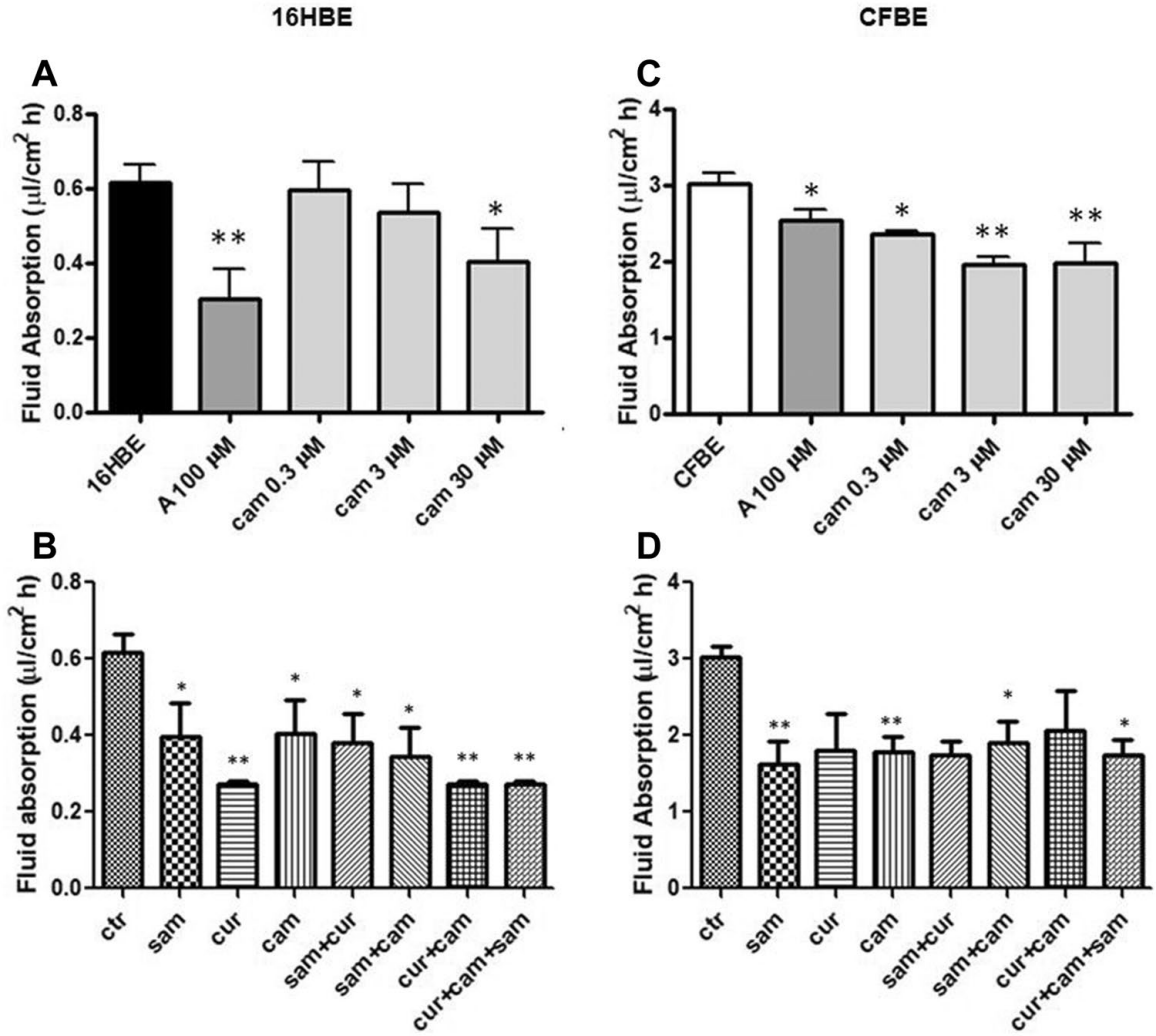
Effect of amiloride, SAM, curcumin and camostat on fluid absorption of 16HBE and CFBE cells. **A** and **B** 16HBE; **C** and **D** CFBE. Statistical significance of differences was evaluated by ANOVA followed by Bonferroni’s post test: *P < 0.05, **P < 0.01 [both for each condition *vs* untreated cells (ctr)]. *A* amiloride, *sam* S-adenosyl methionine, *cur* curcumin, *cam* camostat, *ctr* control (without drugs)

ENaC activity was measured by the surrogate fluid absorption method (see Materials and Methods). We measured the trans-epithelial transport in both immortalized and primary airway epithelial cells. The measurement of in vitro fluid absorption in 16HBE and CFBE cells showed a higher activity in the cells with mutated CFTR (Fig. 2C and 2D) as compared with the corresponding wild-type cells (Fig. 2A and B). This is consistent with the enhanced ENaC activity in CFBE cells. Amiloride significantly reduced the fluid absorption in both 16HBE (Fig. 2A) and CFBE (Fig. 2C) cells, indicating a basal ENaC activity in these cells. Furthermore, the addition of camostat reduced the fluid absorption in a dose-dependent manner, with a significant inhibition at the lowest dose tested of 0.3 μM concentration and the highest inhibition when used at 3 μM and at 30 μM in CFBE (Fig. 2C), and at 30 μM concentration in 16HBE (Fig. 2A).

Treatments with 30 μM camostat, 100 μM SAM, 6 μM curcumin and their combinations generally decreased the monolayers’ fluid absorption in both cell lines (Fig. 2B and D), with the strongest effect provided by curcumin, in respect to SAM and camostat, in 16HBE (Fig. 2B), and a similar effect provided by SAM, curcumin and camostat in CFBE (Fig. 2D).

As shown in the Fig. S1, when amiloride was used (at a concentration of 10 μM) in the presence of camostat, no additive effects were observed in both cell lines, indicating that camostat and amiloride affect ENaC-mediated fluid absorption and that volume change is mediated by ENaC.

These results support the hypothesis that ENaC genes are under epigenetic control and therefore can be modulated by epigenetic modulators, similarly to the anti-protease modulation by camostat. No either synergic or additive effect of double or triple combination was detected.

### Fluid absorption in primary bronchial epithelial cells

To further corroborate our epigenetic and anti-protease hypothesis, primary bronchial epithelial cells, both wild-type and CF (F508del/F508del) were also investigated.

Non-CF and CF primary cells differentiated in ALI conditions and developed a high trans-epithelial electrical resistance after an eight-day culture and a twelve-day culture respectively (Fig. S2A). These data were confirmed by the evaluation of the presence of zonula occludens 1 (ZO-1) protein and acetylated tubulin (differentiated cilia marker) by fluorescence microscopy (Fig. S2B).

**Fig. 3:**
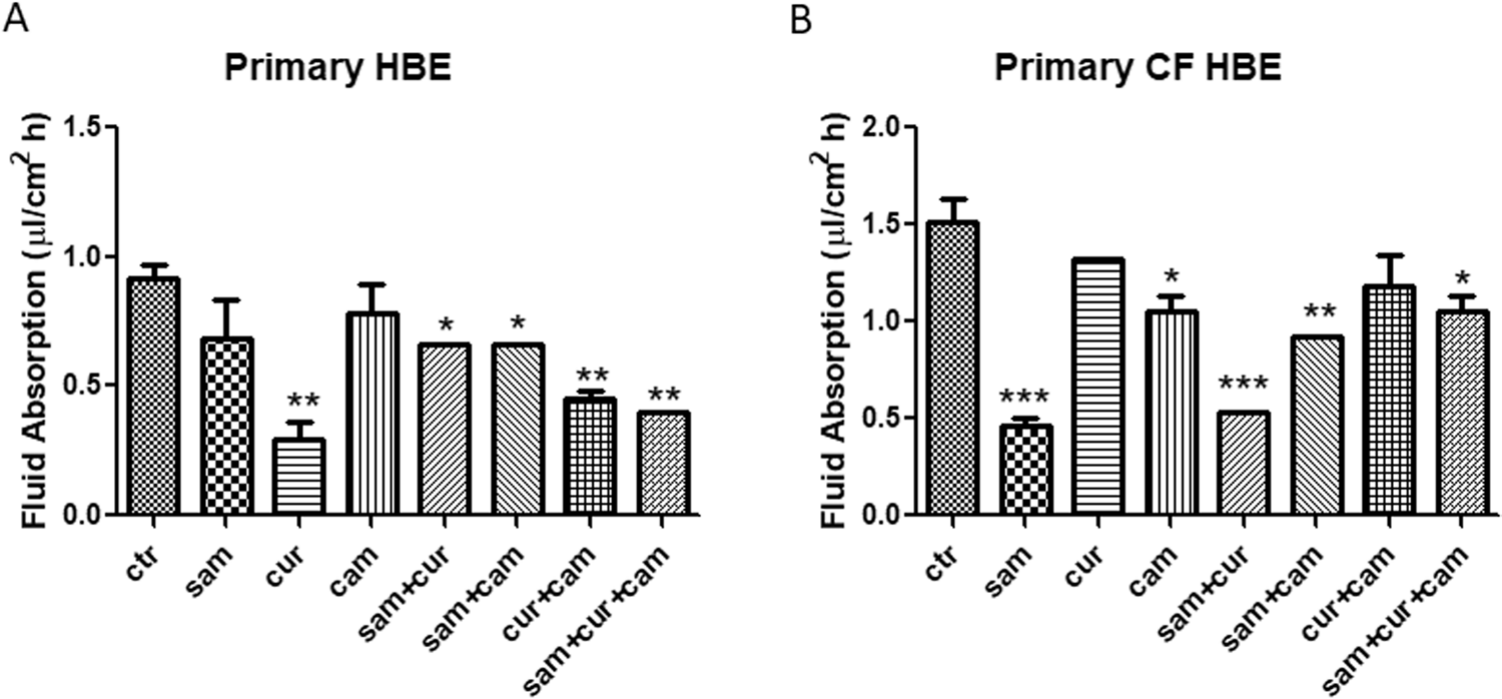
Effect of SAM, curcumin and camostat on fluid absorption of epithelial primary cells both wild-type (on the left) and mutated homozygous F508del (on the right). Statistical significance of differences was evaluated by ANOVA followed by Bonferroni’s post test: *P < 0.05; **P < 0.01, ***P < 0.005 [all for each condition *vs* untreated cells (ctr)]. *sam* S-adenosyl methionine, *cur* curcumin, *cam* camostat

Fluid absorption of non-CF primary cells significantly decreased in the presence of curcumin (alone or in combination with SAM and camostat), whereas single treatments with SAM and camostat resulted ineffective (Fig. 3A). Another significant decrease was observed in the combined treatment with SAM and camostat and in the triple combination of SAM, curcumin and camostat (Fig. 3A). By contrast, the main effect on fluid absorption was exerted by the single treatment with SAM in CF primary cells, leading to a reduction of ENaC activity to about one third compared to untreated cells (Fig. 3B). The strong activity of SAM resulted quenched when used in combination with curcumin and/or camostat, although a significant reduction of fluid absorption remained. Also, camostat (but not curcumin) in single treatment demonstrated the ability to reduce the fluid absorption in CF primary cells (Fig. 3B). Overall, these results led to the possibility of downregulation of ENaC activity by the epigenetic modulation, acting primarily on DNA methylation, as well as by the inhibition of extracellular peptidases. Neither synergistic nor additive activity between epigenetic modulators and camostat was found, suggesting that the modulation of the ENaC gene by epigenetic mechanisms does not allow further modulation of ENaC activity by protease inhibitors*.*

### ENaC and CFTR gene expression in CFBE

Having observed that curcumin and SAM changed the fluid absorption, which is a surrogated measurement of the activity of the ENaC, next we investigated the modulation of transcription of ENaC genes after epigenetic treatments.

**Fig. 4:**
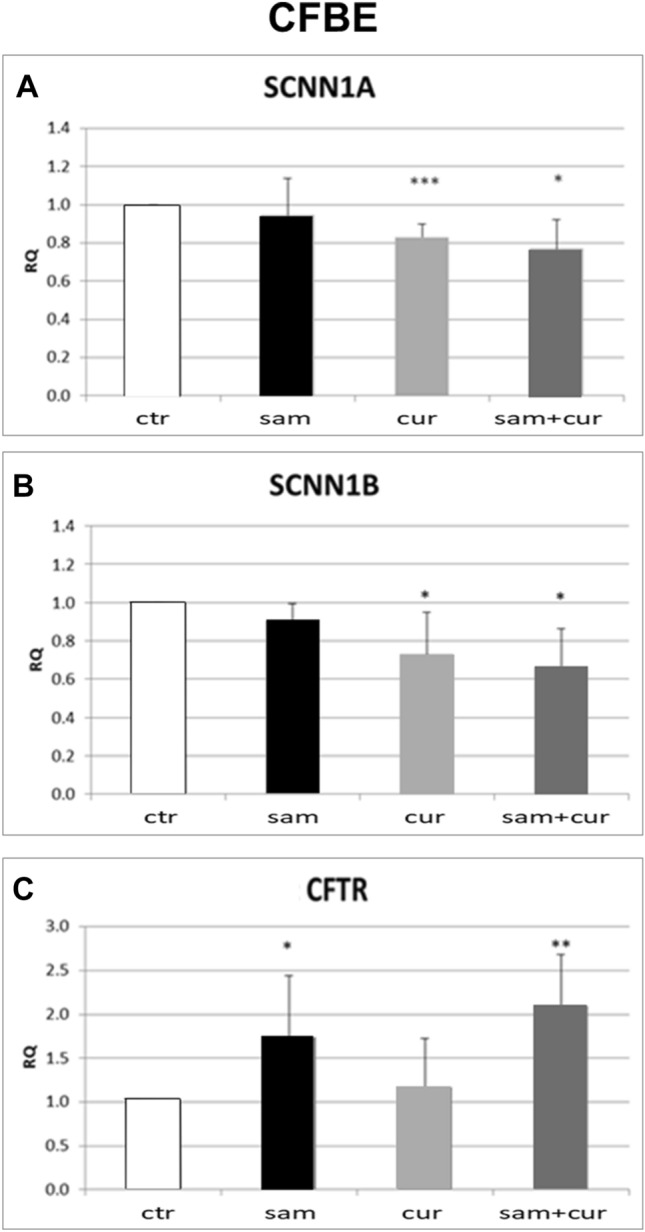
Effect of SAM and curcumin on ENaC and CFTR gene expression in CFBE cells. Statistical significance of differences was evaluated by ANOVA followed by Bonferroni’s post test: *P < 0.05, **P < 0.01, ***P < 0.005 [all for each condition *vs* untreated cells (ctr)]. *sam* S-adenosyl methionine, *cur* curcumin, *RQ* relative quantification

To study the effect of DNA hypermethylation and/or chromatin condensation on the expression of ENaC genes, cells were treated with SAM and/or curcumin, respectively. The modulation of ENaC transcription based on epigenetic approaches was assessed by real-time PCR. In CFBE cell line, treatments appeared to significantly reduce the expression of both *SCNN1A* (Fig. 4A) and *SCNN1B* (Fig. 4B) ENaC subunit genes. In particular, when compared to the control, the averages of the expression reduction resulted as follows: SAM, 7% and 12% reduction for *SCNN1A* and *SCNN1B* respectively; curcumin, 17% and 22% for *SCNN1A* and *SCNN1B* respectively. Interestingly, the combination of both treatments caused a reduction for *SCNN1A* of 21% and for *SCNN1B* of 34%. Of note, whatever treatment did not negatively influence *CFTR* expression, whereas some treatment increased its expression, the highest effect being obtained with SAM (alone or in combination with curcumin) (Fig. 4C). This may result from a complex equilibrium between the epigenetic effect exerted by drugs and CFTR–ENaC interactions, as well as from a possible effect on other genes with a modulatory effect on CFTR transcription. In the 16HBE cell line (Fig. S3), similar results were obtained for *SCNN1A* gene, with a reduction of expression up to 22% after SAM and curcumin combined treatment. The expression of the *SCNN1G* in CFBE and that of both *SCNN1B* and *SCNN1G* in 16HBE, could not be analyzed due to their very low expression, at the limit of detection.

### ENaC and CFTR gene expression in CF primary bronchial epithelial cells

**Fig. 5:**
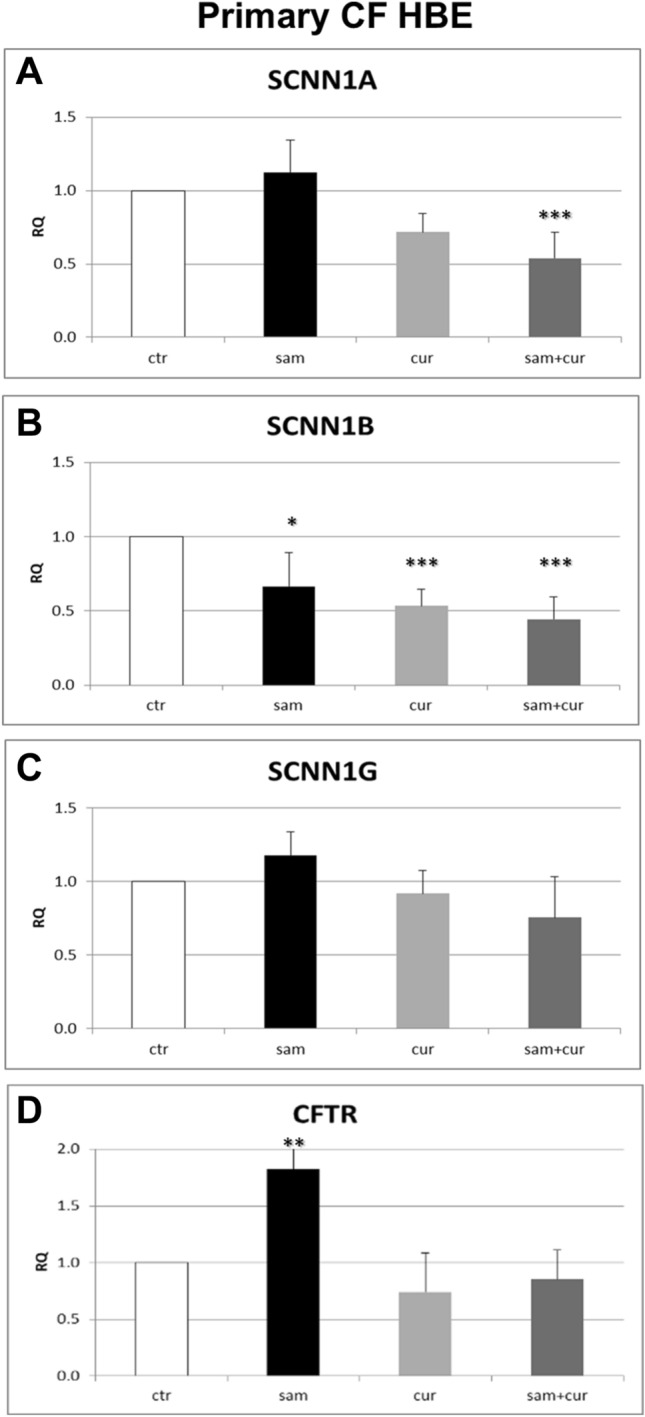
Effect of SAM and curcumin on ENaC and CFTR gene expression in CF primary bronchial epithelial cells. Statistical significance of differences was evaluated by ANOVA followed by Bonferroni’s post test: *P < 0.05, **P < 0.01, ***P < 0.001 [all for each condition *vs* untreated cells (ctr)]. *sam* S-adenosyl methionine, *cur* curcumin, *RQ* relative quantification

Corresponding experiments were performed in CF primary cells. The results showed significant differences in the expression of both *SCNN1A* and *SCNN1B* genes after SAM, curcumin and SAM and curcumin combined treatments (Fig. 5).

In particular, *SCNN1A* gene expression was reduced after treatment with curcumin of about 30% and a reduction of about 50% was shown after treatment with SAM and curcumin in combination (Fig. 5A). *SCNN1B* gene expression was reduced after treatment with SAM, curcumin and with SAM and curcumin in combination of about 30%, 50% and 60% respectively (Fig. 5B). On the contrary, the results obtained for *SCNN1G* gene did not allow to evidence any significant difference of gene expression after epigenetic treatments (Fig. 5C). Thus, also in primary homozygous CF (F508del/F508del) cells SAM and/or curcumin treatments decrease the expression of both *SCNN1A* and *SCNN1B* gene, suggesting a possible DNA hypermethylation/chromatin condensation effect by these drugs. Besides the immortalized cells, at least one epigenetic treatment increased CFTR gene expression also in CF primary cells (Fig. 5D). CFTR expression increased after SAM treatment of about 80% in respect to control.

### DNA methylation of SCNN1B gene promoter in CFBE

Given that promoter DNA methylation is a well-established mechanism of epigenetic gene silencing and the most significant modulation of expression were obtained for *SCNN1B* gene, methylation analysis of *SCNN1B* promoter was performed by bisulfite sequencing. To demonstrate a correlation between the onset of a hypermethylated pattern and the decreased gene expression, the DNA methylation pattern of the gene controlling regions was investigated in CFBE and CF primary bronchial epithelial cells.

A preliminary analysis of overall *SCNN1B* methylation (CpG + non-CpG, in both the pre-island and the island zones; Fig. S4) in CFBE revealed a statistically significant hypermethylation level induced by SAM treatment. In particular, after SAM treatment CFBE showed a total methylation level of 11.2%, whereas in control the total methylation resulted to be of 6.8%, comparable to curcumin treatment (6.5%). A slight increase to 7.5% of total methylation after SAM and curcumin combined treatments was evidenced, although not statistically significant.

**Fig. 6:**
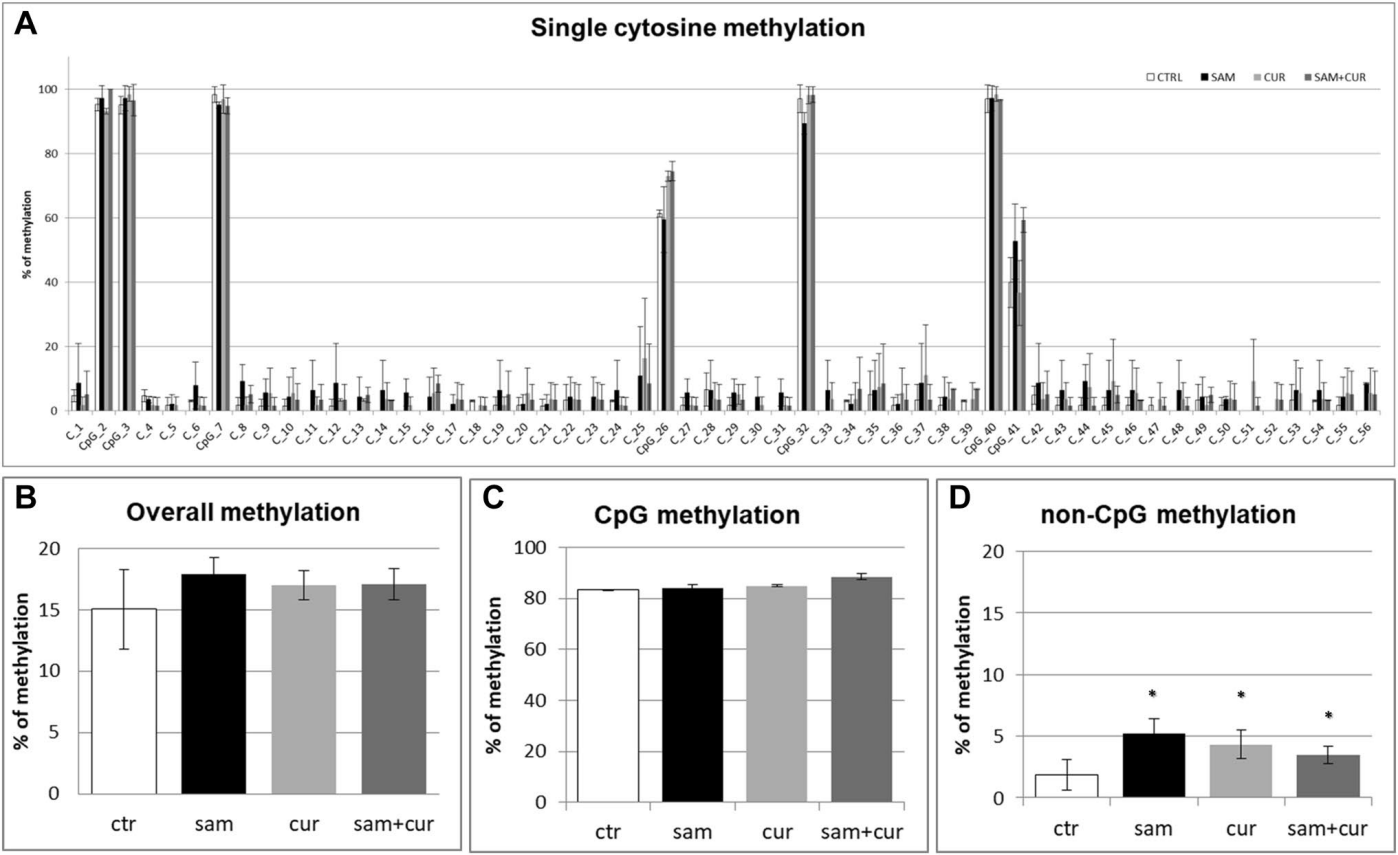
Effect of SAM and curcumin on methylation of the SCNN1B pre-island zone in CFBE. **A** Results at the level of single cytosine. **B** Overall (CpG + non-CpG) DNA methylation. **C** Cumulative results at the level of CpG cytosines. **D** Cumulative results at the level of non-CpG cytosines. Statistical significance of differences was evaluated by a contingency table and *χ*^2^ test in (**A**) n.s. (also if CpG e non-CpG were analyzed separately) and by ANOVA followed by Bonferroni’s post test in (**B**) n.s., **C** n.s. and **D** *P < 0.05 (each condition *vs* untreated cells (ctr)). *sam* S-adenosyl methionine, *cur* curcumin

The pre-island zone contains 7 CpG sites and 49 non-CpG sites (Fig. 6A). A single-site analysis of this zone in CFBE, revealed that the CpG sites were almost completely methylated (from 40.0% to 98.3%) and, consequently, no treatment significantly changed their methylation status. Only in the 41st CpG moiety, whose status is less methylated (< 40%), SAM alone and SAM and curcumin combination increased the methylation status. Remarkably, near all non-CpG sites appeared to increase their methylation level after epigenetic treatments. Thirty-nine (out of 49) non-CpG moieties of the pre-island zone resulted more methylated than the control after treatment with SAM alone and SAM and curcumin combination (although, probably due to high variability, it resulted not possible to demonstrate a statistical significance, at single moiety level). In the pre-island zone, all the treatments induced a slight increment of overall (CpG + non-CpG) DNA methylation, although not statistically significant (Fig. 6B). The difference in methylation level of the unique modulated CpG site of the pre-island zone is not evident if cumulative CpG methylation is taken into account (Fig. 6C). The cumulative non-CpG sites of the pre-island zone showed a variable methylation level, although they resulted generally much less methylated than CpG sites, with a statistically significant hypermethylation after all the treatments (Fig. 6D).

**Fig. 7:**
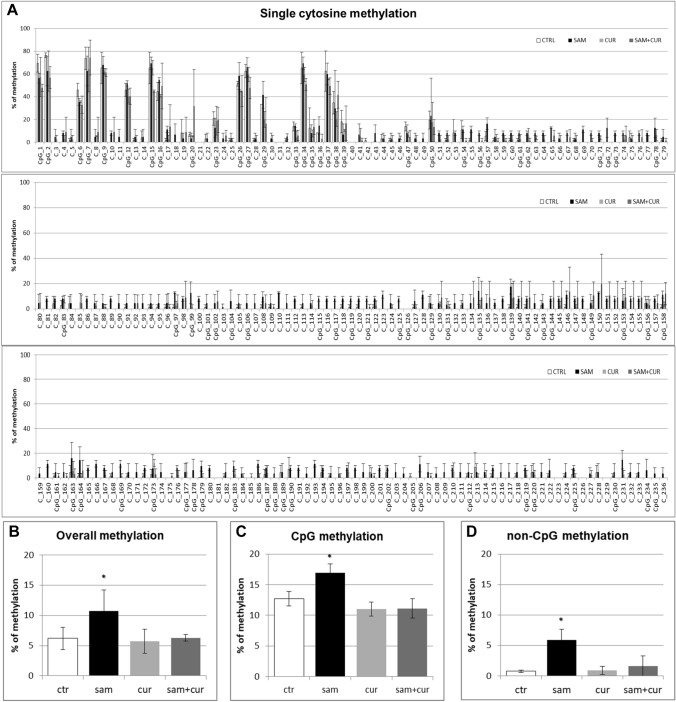
Effect of SAM and curcumin on methylation of the SCNN1B island zone in CFBE. **A** Results at the level of single cytosine. **B** Overall (CpG + non-CpG) DNA methylation. **C** Cumulative results at the level of CpG cytosines. **D** Cumulative results at the level of non-CpG cytosines. Statistical significance of differences was evaluated by a contingency table and *χ*^2^ test in A) *P *< 0.0001 (overall and also if CpG (*P *< 0.0001) e non-CpG (*P *< 0.0001) were analyzed separately) and by ANOVA followed by Bonferroni’s post test in (**B**, **C** and **D**) *P < 0.05 (for all, sam *vs* untreated cells (ctr)). *sam* S-adenosyl methionine, *cur* curcumin

The island zone contains 78 CpG sites, 158 non-CpG sites and, among the latter, 3 CCTCC elements (Fig. 7A). The CCTCC is a new structural epigenetic element recently proposed to have a distinct and relevant functional role in the context of non-CpG methylation [61]. The first 20 CpGs (up to CpG_39) at the beginning of the island zone resulted partially methylated and the treatments did not appear to increase the methylation levels except for few sites (CpG_16, CpG_20, CpG_29, CpG_36 and CpG_38). Methylation status resulted increased (in a statistically significant manner, at single moiety level) in near all the remaining CpG and non-CpG cytosines after SAM and, in some cases, also after SAM and curcumin combined treatment. In the island zone, overall (CpG + non-CpG) DNA methylation level resulted unchanged after all treatments, except for a statistically significant increase after SAM treatment (Fig. 7B). A slight and statistically significant hypermethylating effect, after SAM treatment is visible for both CpG (Fig. 7C) and non-CpG (Fig. 7D) moieties.

**Fig. 8:**
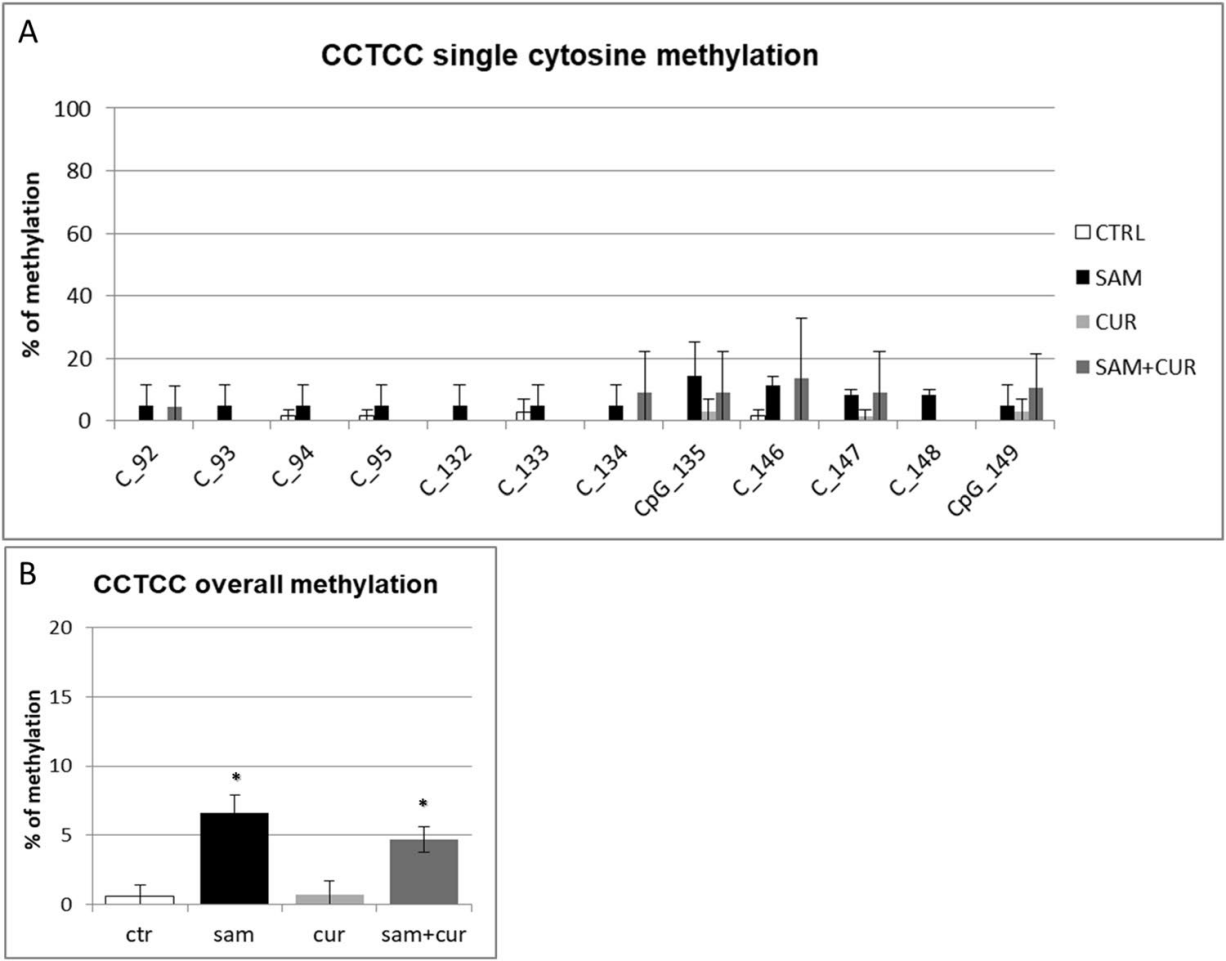
Effect of SAM and curcumin on methylation of the SCNN1B CCTCC elements of the island zone in CFBE. **A** Results at the level of single cytosine. **B** Cumulative results of all cytosines. Statistical significance of differences was evaluated by a contingency table and *χ*^2^ test in (**A**) n.s. and by ANOVA followed by Bonferroni’s post test in (**B**) *P < 0.05 [each condition *vs* untreated cells (ctr)]. *sam* S-adenosyl methionine, *cur* curcumin

Among the non-CpG methylation, the methylation status of cytosines within the three CCTCC elements was analyzed (Fig. 8). A slight increase in the methylation levels after SAM alone and SAM and curcumin combined treatments is evident for all the 12 cytosines included in these elements (Fig. 8A) (although, probably due to high variability, it resulted not possible to demostrate a statistical significance, at single moiety level). Also the cumulative effect is well visible and statistically significant (Fig. 8B).

### DNA methylation of SCNN1B gene promoter in CF primary bronchial epithelial cells

Also in CF primary bronchial epithelial cells, a preliminary analysis of overall methylation (CpG + non-CpG, in both pre-island and island zones; Figure S5) was done. SAM treatment induced a statistically significant increase in overall methylation level, up to 23.3% in respect to control at 2.2%, whereas no increase was evidenced after curcumin (2.4%) and SAM and curcumin combined (2.1%) treatments.

**Fig. 9:**
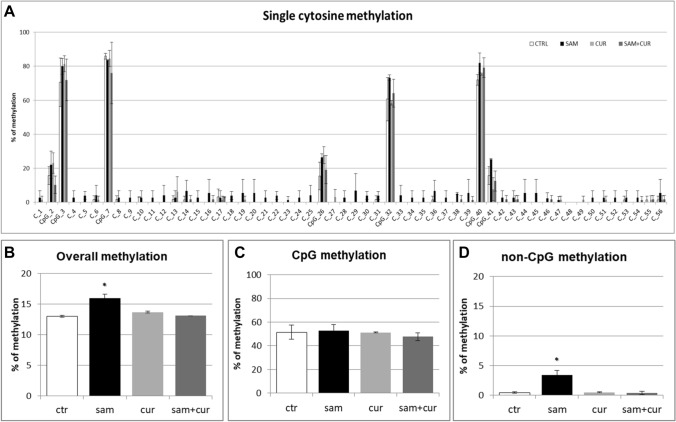
Effect of SAM and curcumin on methylation of the SCNN1B pre-island zone in CF primary bronchial epithelial cells. **A** Results at the level of single cytosine. **B** Overall (CpG + non-CpG) DNA methylation. **C** Cumulative results at the level of CpG cytosines. **D** Cumulative results at the level of non-CpG cytosines. Statistical significance of differences was evaluated by a contingency table and *χ*^2^ test in A) *P *< 0.0001 [overall and also for non-CpG (*P *< 0.001), but not for CpG (n.s.)] and ANOVA followed by Bonferroni’s post test in (**B**) *P < 0.05, **C** n.s. and **D** *P < 0.05 [for sam *vs* untreated cells (ctr)]. *sam* S-adenosyl methionine, *cur* curcumin

All the pre-island CpG sites were methylated (from 15.5% to 86.0%; Fig. 9A), with no treatment that significantly changed their methylation status. By contrast, the non-CpG sites showed a completely demethylated status that changed, in a statistically significant manner, after SAM treatment to a slight methylation of 45 (out of 49) non-CpG moieties (from 1.4% to 7.0%; Fig. 9A). The analysis of overall methylation of the pre-island zone (Fig. 9B) showed a statistically significant increase of methylation after SAM treatment. The absence of a global effect on the cumulative CpG moieties (Fig. 9C) and a statistically significant hypermethylation after SAM treatment on the cumulative non-CpG moieties (Fig. 9D) were evident.

**Fig. 10:**
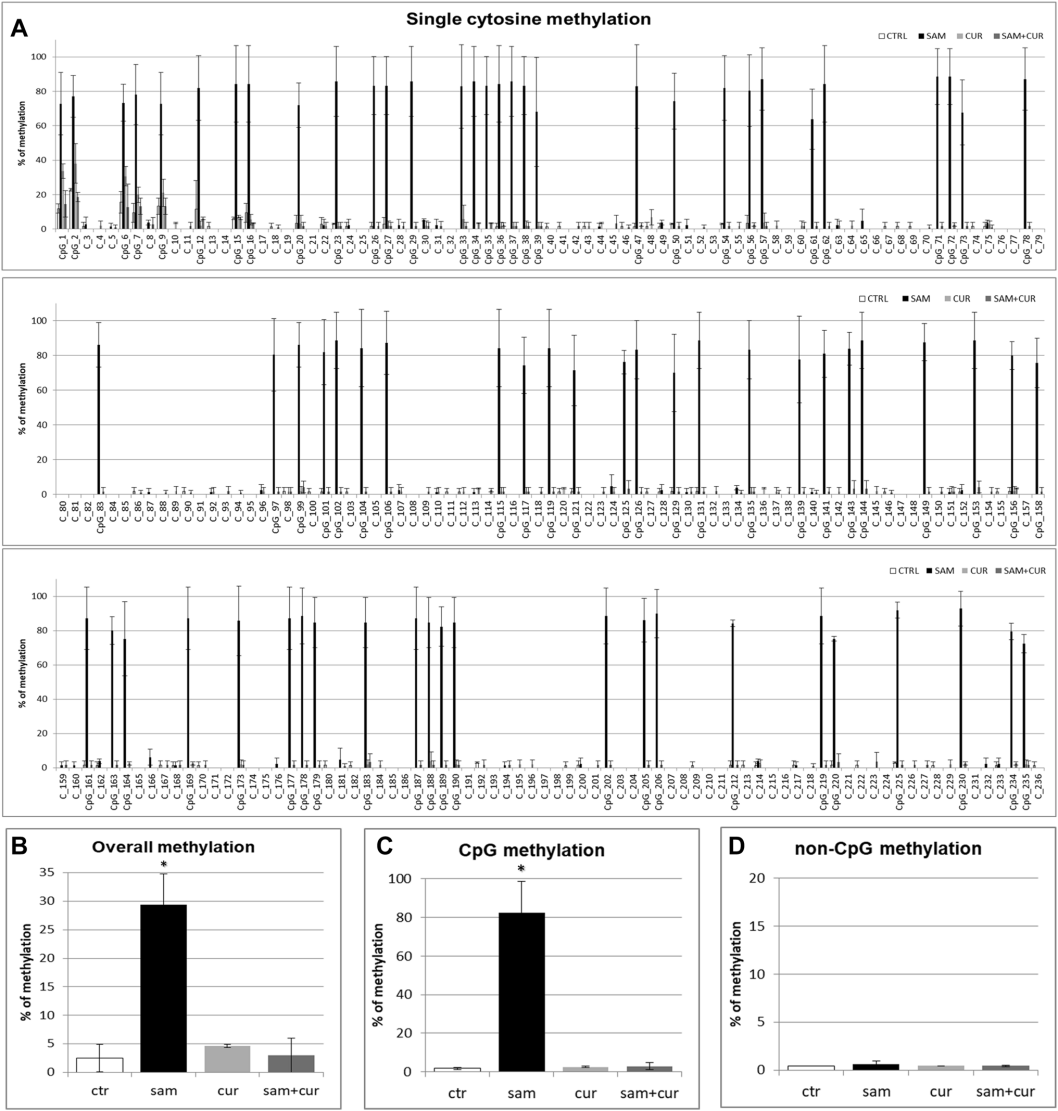
Effect of SAM and curcumin on SCNN1B CpG island zone methylation in CF primary bronchial epithelial cells. **A** Results at the level of single cytosine. **B** Overall (CpG + non-CpG) DNA methylation. **C** Cumulative results at the level of CpG cytosines. **D** Cumulative results at the level of non-CpG cytosines. Statistical significance of differences was evaluated by a contingency table and *χ*^2^ test in (**A**) *P *< 0.0001 [overall and also for CpG (*P *< 0.0001), but not for non-CpG (n.s.)] and ANOVA followed by Bonferroni’s post test in (**B**) *P < 0.01, **C** *P < 0.01 (for sam *vs* untreated cells (ctr)) and **D** n.s. *sam* S-adenosyl methionine, *cur* curcumin

In the island zone of CF primary cells (Fig. 10A) only 25 (out of 78) CpG cytosines showed a basal level of methylation (with only the first 8 CpG cytosines with a noticeable level). SAM treatment induced a very large, and statistically significant, increase (to 82.3% on average) of the methylation status of all the 78 CpG sites. Consequently, the total methylation level showed a large and statistically significant increment after SAM treatment, to about 29% in respect to control at 2.5% (Fig. 10B). This mainly depends on overall CpG moieties (Fig. 10C), as overall non-CpG moieties do not contribute, having no (or barely detectable) methylation not incremented by treatments (Fig. 10D).

**Fig. 11:**
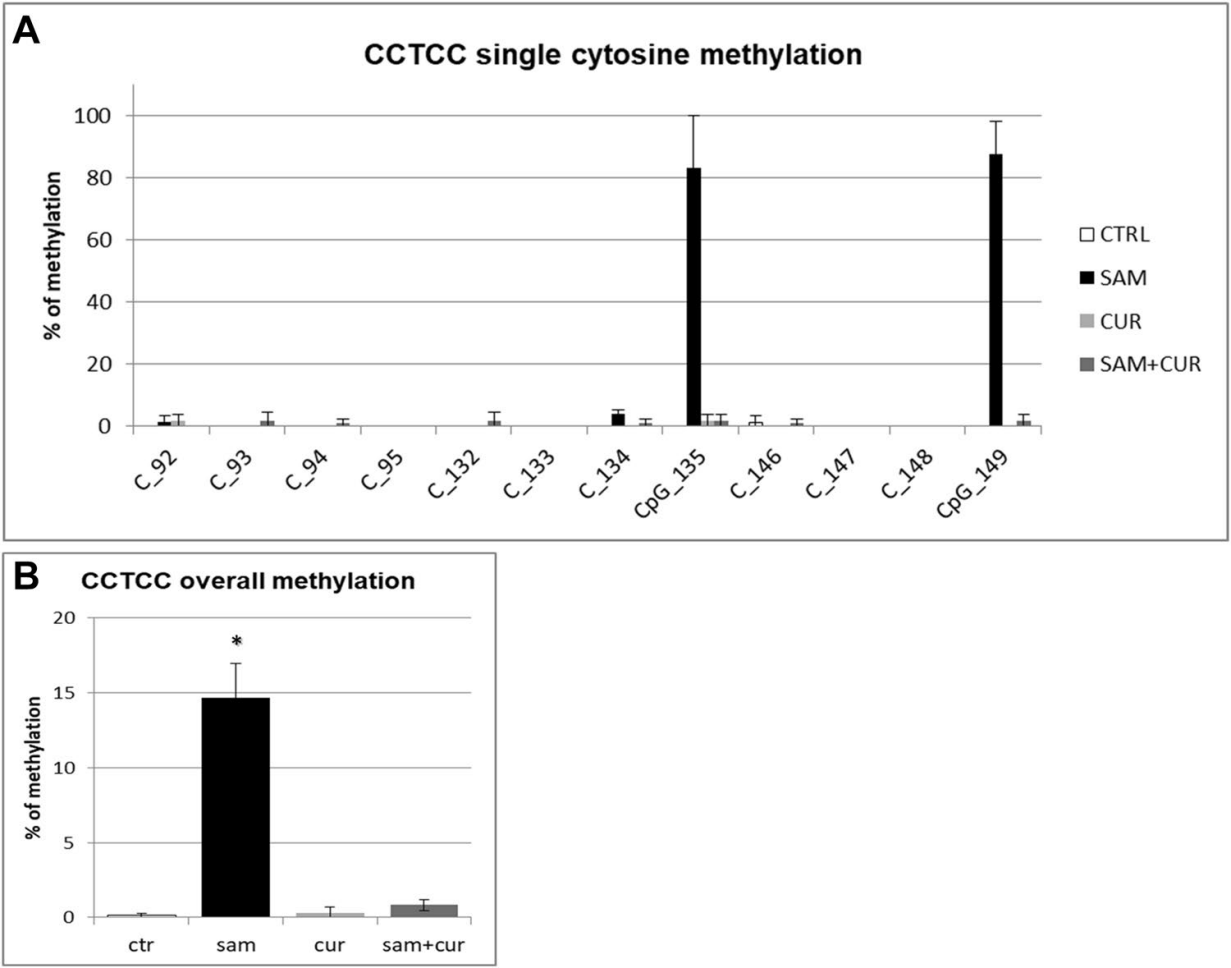
Effect of SAM and curcumin on methylation of the SCNN1B CCTCC elements of the island zone in CF primary bronchial epithelial cells. **A** Results at the level of single cytosine. **B** Cumulative results of all cytosines. Statistical significance of differences was evaluated by a contingency table and *χ*^2^ test in (**A**) *P *< 0.0001 and by ANOVA followed by Bonferroni’s post test in (**B**) *P < 0.001 [sam *vs* untreated cells (ctr)]. *sam* S-adenosyl methionine, *cur* curcumin

Finally, the analysis of the methylation status of cytosines within the three CCTCC elements of island zone was performed. As observed in Fig. 11A, these elements showed no basal methylation in the control and a considerable, statistically significant, increase in the methylation levels after SAM treatment in two cytosines (CpG_135 and CpG_149). Overall (Fig. 11B), SAM treatment resulted in a statistically significant increase of methylation of cytosines within these elements to 14.7%.

## Discussion

Although CF is caused by mutations in the *CFTR* gene, the hallmarks of the CF lung disease are the deficient CFTR-mediated chloride secretion associated with increased ENaC-mediated fluid absorption. This leads to airway surface dehydration and ensuing pathophysiologic consequences, such as opportunistic bacterial infections. In this context, proteolytic activation of ENaC by neutrophilic elastase and other proteases may be not only involved in the first steps of CF lung disease but also a key mechanism aggravating airway surface dehydration in CF airways [70]. Thereby, a strategy to treat CF lung disease is to inhibit protease activation of ENaC [10, 35, 45]. In particular, since ENaC is positively regulated by channel-activating proteases (CAPs), CAP inhibitors are predicted to be beneficial in diseases associated with impaired mucociliary clearance. The investigation of CAP inhibitors is indeed actively pursued [71]. Camostat is a low-molecular weight inhibitor of extracellular peptidase involved in ENaC activation that potently attenuated amiloride-sensitive sodium transport, in non-CF and CF primary bronchial epithelial cells and in an animal model [44]. It is already in clinical use as a trypsin-like serine protease inhibitor for the treatment of pancreatitis and postoperative reflux esophagitis [72, 73].

On the other hand, another way to reduce ENaC activity may be its transcriptional downregulation, possibly obtained acting at chromatin level, directly or by DNA methylation modulation. The role of epigenetics in CF is still poorly understood, although DNA methylation and histone modification have been implicated in CFTR gene regulation [74]. For example, a recent study provides evidence that histone deacetylase (HDAC) inhibitors determine functional correction of Class II and III CFTR variants, restoring cell surface chloride channel activity in primary human bronchial epithelial cells [75]. Epigenetic regulation of ENaC has been investigated at the level of collecting ducts in the kidney [76], while no extensive studies have been conducted in the airways. We have recently shown that *SCNN1A*, *SCNN1B*, and *SCNN1G* genes are actually regulated in their transcription by epigenetic mechanism, such as DNA methylation, in airway epithelial cells [56]. Here, speaking at direct chromatin level, we have employed curcumin, a polyphenol natural product isolated from the rhizome of the plant *Curcuma longa*, and a common dietary compound, that is a selective histone acetyltransferase (HAT) inhibitor since it specifically represses the P300/CREB-binding protein, a member of HAT family, inducing chromatin condensation [77]. It has also been demonstrated that it may exert its positive (from CF point of view) effects by altering the concentration of free calcium in the endoplasmic reticulum lumen, thus influencing the capacity of calcium-dependent chaperone mechanisms to retain the misfolded F508del CFTR protein, that could be delivered to the plasma membrane [78]. At DNA methylation level, S-adenosyl methionine (SAM) is a co-substrate involved in methyl group transfer to DNA cytosines [64], with consequent DNA hypermethylation [79, 80]. It is already used in clinics as a therapeutics for multiple neuropsychiatric conditions [81].

According to our results, the fluid absorption was greater in untreated CFBE (Fig. 2C and D) than 16HBE (Fig. 2A and B) and in primary CF HBE (Fig. 3B) than in wild-type HBE (Fig. 3A), reflecting the expected effect of a dysfunctional CFTR on ENaC. Fluid absorption resulted to be reduced after treatment by camostat, SAM, curcumin and combined treatments in CFBE (Fig. 2C and D), 16HBE (Fig. 2A and B), primary CF HBE (Fig. 3B) and wild-type HBE (Fig. 3A). The greatest effect of reduction on fluid reabsorption was shown by SAM and SAM combined with curcumin in primary CF HBE (Fig. 3B). These treatments had a great quantitative effect also in CFBE (Fig. 2D), although in these cells also the other treatments induced a quantitatively similar reduction. These data strongly indicate that primary airway epithelial cells grown at air–liquid interface, which mimic at best the physiological asset of the airway epithelium in vivo [82], respond to the epigenetic modulators and should be further investigated in this context.

Overall, both SAM and curcumin (and their combination) revealed to be able to downregulate the *SCNN1A* and *SCNN1B* gene expression in both CFBE (Fig. 4A and B) and primary CF HBE (Fig. 5A and B). The best possibility of expression inhibition for both genes was however shown in primary CF HBE. In these cells, a clear and significant effect of expression inhibition of *SCNN1B* gene was shown by both SAM and curcumin, with the greatest quantitative effect (with more than 50% of expression inhibition) by their combined treatment (Fig. 5B). The combined treatment produced a quantitatively similar expression inhibition also for *SCNN1A* gene (Fig. 5A). These data underlie that the effect on fluid absorption might be due to a reduced ENaC transcriptional expression exerted by SAM/curcumin and further support the primacy of primary airway epithelial cells in evaluating epigenetic modulators in the CF context.

Overall methylation of *SCNN1B* gene in primary CF HBE (Fig. S5) resulted to be lower than that of CFBE (Figure S4), with a greatest responsiveness of primary CF HBE to SAM treatment that, in these cells, induced a greater hypermethylation (to 23.3%) than in CFBE (to 11.2%). A lower overall methylation of primary CF HBE in respect to CFBE were evidenced, at level of individual cytosines as well as of cumulative CpG and non-CpG moieties, in both pre-island (Fig. 9 as compared to Fig. 6, respectively) and island (Fig. 10 as compared to Fig. 7, respectively) zones. The overall high responsiveness to SAM treatment of primary CF HBE appeared to be almost entirely sustained by the hypermethylation of CpG sites of the island zone (Fig. 10C), with a huge effect (to more than 80% of methylation), although an effect could be also seen at level of non-CpG sites of the pre-island zone (Fig. 9D). The overall responsiveness to SAM treatment of CFBE appeared to depend on non-CpG sites of pre-island zone (Fig. 6D) and on both CpG and non-CpG sites of island zone (Fig. 7C and D, respectively). The CCTCC elements of the island zone resulted completely non-methylated in untreated primary CF HBE  cells (Fig. 11) and with very low level of methylation of only some cytosines in untreated CFBE (Fig. 8). High level of methylation could be induced by SAM on specific cytosines (CpG_135 and CpG_149) in CF primary cells, whereas a lower level of methylation could be induced in a wider number of cytosines in CFBE (also by the combined SAM and curcumin treatment). With the exception of cytosines CpG_135 and CpG_149 (island zone) in CF primary cells, and cytosines CpG_41 (pre-island zone) and CpG_16, CpG_20, CpG_29, CpG_36, CpG_38 (island zone) in CFBE, which showed a peculiar modulatory pattern, no other single moiety showed a significant individual modulatory behavior. It is remarkable that the two CpG_135 and CpG_149 cytosines belong to an epigenetic elements (the CCTCC) already described as a non-CpG structural component with possible functional role distinct to that of CpG methylation [61]. In the *SCNN1B* control regions analyzed, rather than a role for single cytosines, what seems to be prevalent is a different behavior of distinct structural elements (the CpG, non-CpG and CCTCC) which may have different functional roles. As previously proposed for *myogenin* gene in muscle [61, 83], the non-CpG moieties, and in particular some CpC-rich elements (such as the CCTCC), may have the role of priming the demethylation of a wider DNA region during transcriptional activation. Obviously, further experiments allowing the dynamic, over time (for example during differentiation or after a stimulus), assessment of DNA methylation patterns are needed to verify this hypothesis also for *ENaC* genes in respiratory epithelium. However, it could be speculated that the tendency to a hypermethylation of non-CpG moieties after experimental manipulation, may evidence their peculiar capacity of a rapid modulation of their methylation pattern. All in all, hypermethylated patterns determined by SAM may explain the lower expression of *SCNN1B* gene and hence also the reduction in fluid absorption.

Interestingly, the limited effect obtained in wild-type primary HBE with SAM and camostat (Fig. 3A) seems to suggest that in non-CF cells hypermethylation of ENaC genes and proteolytic activation of ENaC protein are not sufficient to have significant downstream physiologic effects on fluid absorption. On the contrary, the large effect exerted in these cells by curcumin may depend on its dual action on ENaC gene expression inhibition and CFTR trafficking enhancement. Although with significant quantitative differences, this effect is also partially replicated in 16HBE (Fig. 2B). On the other hand, in primary CF HBE a common effect of a significant reduction of fluid absorption by SAM but not by curcumin can be seen (Fig. 3B). This effect may be explained by the increase in *CFTR* expression exerted by SAM but not by curcumin (Fig. 5D) additional to the repression of expression of *SCNN1B* gene (Fig. 5B). Also this effect is replicated (also in this case with some differences) in CFBE (Figs. 2D and 4). These results indicate that fluid absorption in CF cells is conditioned by a complex interplay distinct from that of non-CF cells. However, in both CF and non-CF cells, *ENaC* and *CFTR* gene expression and interplay, as well as the overall fluid absorption, seem to depend on epigenetic modulation.

It is interesting to consider that SAM and curcumin act at the transcription level, while camostat exerts its activity at protein level. This could explain the lack of additive effect, with SAM and curcumin reducing the expression of the protein to a level that makes the effect of camostat hardly detectable.

Overall, our results suggest that the function of ENaC, as evaluated by fluid absorption assay, may be downregulated by epigenetic modulation, with approaches acting on both DNA methylation and chromatin condensation, as well as by activity attenuation by inhibiting extracellular peptidase. In particular at epigenetic level, the suitable targets for an effective expression downregulation appeared to be both *SCNN1A* and *SCNN1B* genes. However, no synergistic or additive actions were found between epigenetic modulators and camostat, suggesting that the downregulation of ENaC subunits by epigenetic mechanisms does not allow a further decrease of ENaC function by protease inhibitors, and viceversa.

The fact that epigenetic treatments did not negatively influence *CFTR* expression, but even SAM treatment increased its expression in both CFBE and CF primary cells, reasonably excludes the undesirable effect of worsening any residual activity that CFTR could possibly retain even if mutated. On the other hand, a suppression effect on other genes was not explored and cannot be excluded only on the basis of the results about *CFTR*. Future experimental evaluation of possible “off-target” effects of these treatments are mandatory before a therapeutic application to CF. However, it should be taken into account that both SAM and camostat are already approved as therapeutics for other pathologies and that curcumin is a common non-toxic dietary compound. The problem of possible collateral damage due to a dysregulation of the expression of other genes has been already evaluated and solved in other pathological contexts. Mostly, the applicability of these treatments depends on the possibility of their moving from other pathologies to CF. The topic is that of drug repositioning, a field with increasing importance also in CF.

In conclusion, the possibility to reduce ENaC activity using drugs already approved as therapeutics for other pathologies or a non-toxic dietary compound, with no repression or even enhancement of *CFTR* expression, open new possibilities for CF treatment. In particular, the possibility arises of using this epigenetic approach additionally to present modulatory therapies for CF, or as an alternative in case of their ineffectiveness as, for example, on not responding CFTR mutated genotypes.

### Supplementary Information

Below is the link to the electronic supplementary material.

Supplementary file1 (PDF 672 KB)

## Data Availability

Data and material are available on request from the authors.
