# Supplementary material for: Downregulation of epithelial sodium channel (ENaC) activity in cystic fibrosis cells by epigenetic targeting
Source: Cell Mol Life Sci. 2022 Apr 25;79(5):257. doi: 10.1007/s00018-022-04190-9 (PMC9035428; doi:10.1007/s00018-022-04190-9)
Supplement: Supplementary file 1 — Supplementary file1 (PDF 672 KB) [file 18_2022_4190_MOESM1_ESM.pdf]

SUPPLEMENTARY MATERIALS

SUPPLEMENTARY FIGURES

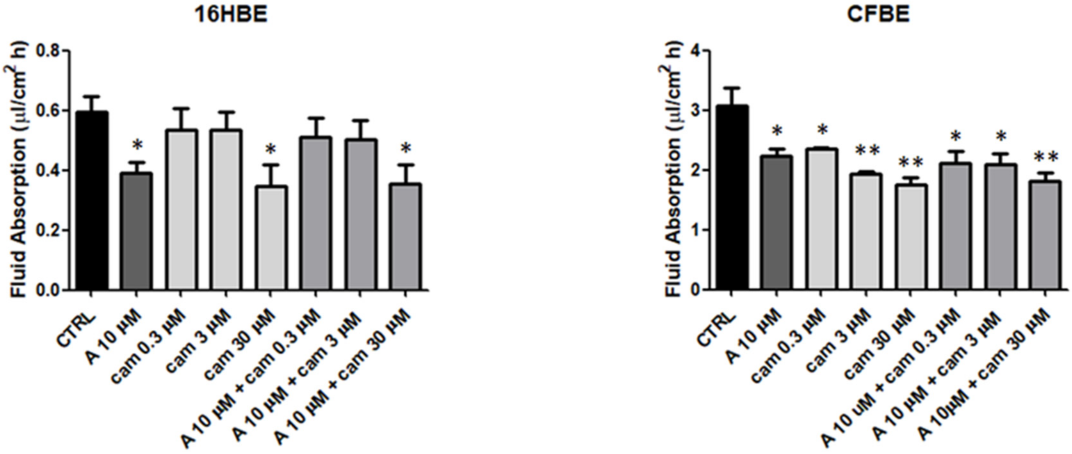

Figure S1 – Combined effect of amiloride and camostat on fluid absorption in 16HBE and CFBE cells.
Statistical significance of differences was evaluated by ANOVA followed by Bonferroni's post test: *P<0.05; **P<0.01, (all for each condition *vs* untreated cells (CTRL)). A = amiloride; cam = camostat.

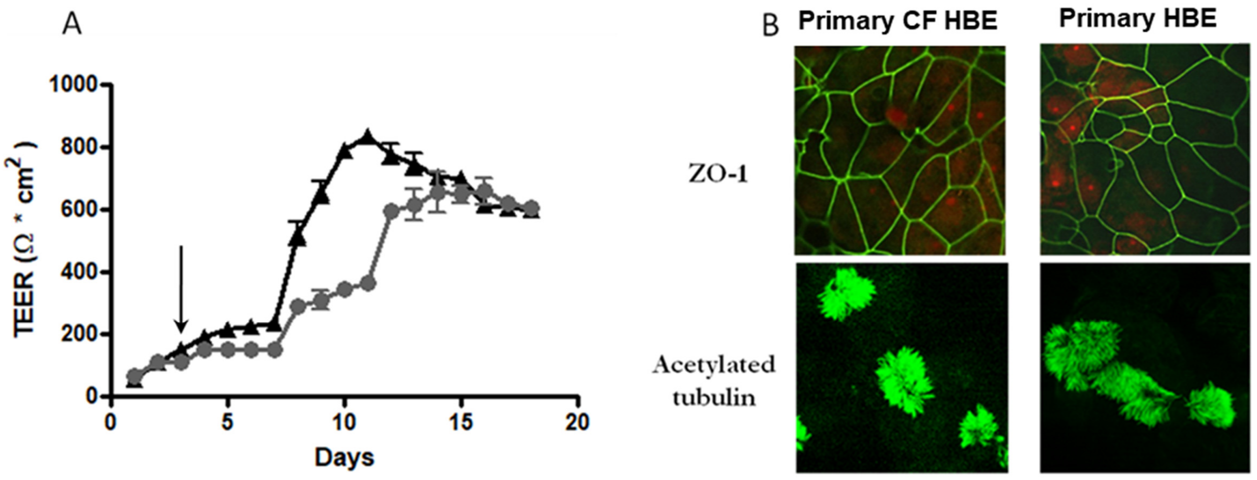

Figure S2 - Epithelial differentiation assessment. Primary airway epithelial cells were cultured at the air-liquid interface (ALI) and evaluated for transepithelial electrical resistance (TEER) and zonula occludens 1 (ZO-1) protein and acetylated tubulin expression. **A)** TEER of primary cells grown on Snapwells was measured daily. From day 3 of culture (indicated by an arrow), cells were grown on ALI condition. Black triangles = wild type primary cells. Grey circles = CF primary cells. **B)** Primary cells were stained for ZO-1 and acetylated tubulin after 15 days of culture on ALI condition on Snapwells (overall 18 days of culture). Both primary cells showed a typical chicken-wire pattern of ZO-1 expression (green signal, top panels) and expression of acetylated tubulin (green signal, bottom panels), a marker of ciliated cells differentiation. For the ZO-1 staining, nuclei were counterstained with propidium iodide. Images were obtained with an original magnification of 60X.

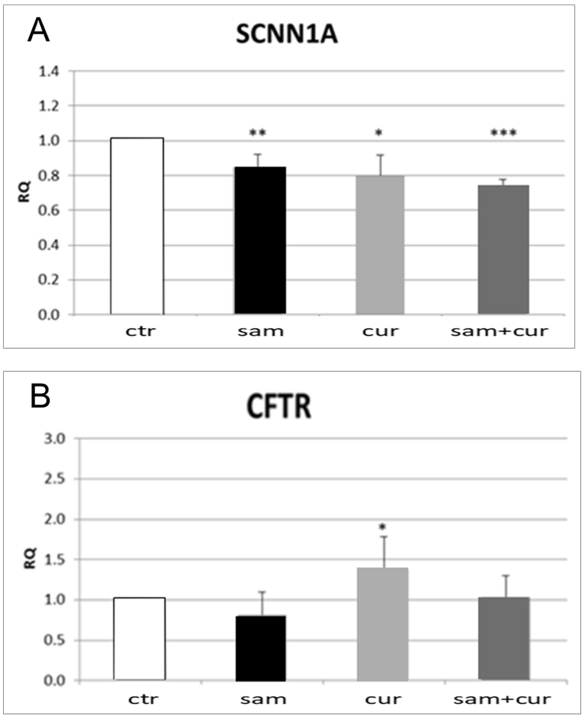

Figure S3 – Effect of SAM and curcumin on ENaC and CFTR gene expression in 16HBE cells.
Statistical significance of differences was evaluated by ANOVA followed by Bonferroni's post test: *P<0.05; **P<0.01; ***P<0.005 (all for each condition *vs* untreated cells (ctr)). sam = S-adenosyl methionine, cur = curcumin, RQ = relative quantification.

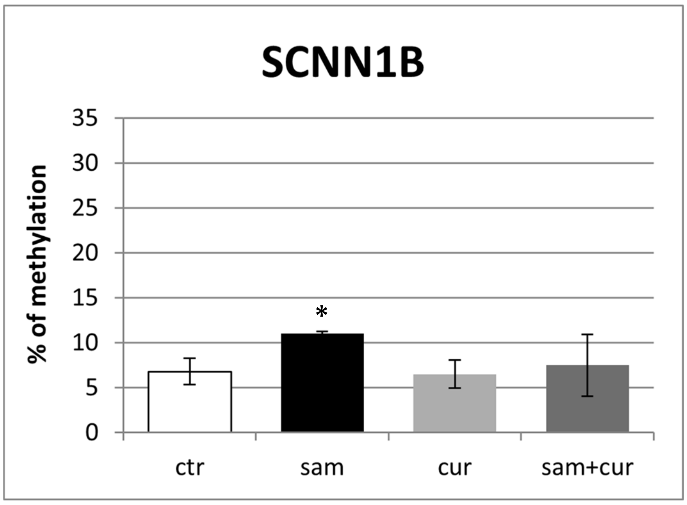

Figure S4 – Overall (CpG + non-CpG) DNA methylation of SCNN1B gene in CFBE cells, with the effect of SAM and curcumin on pre-island and island zones taken together.
Statistical significance of differences was evaluated by ANOVA followed by Bonferroni's post test: *P<0.05 (for sam *vs* untreated cells (ctr)). sam = S-adenosyl methionine, cur = curcumin.

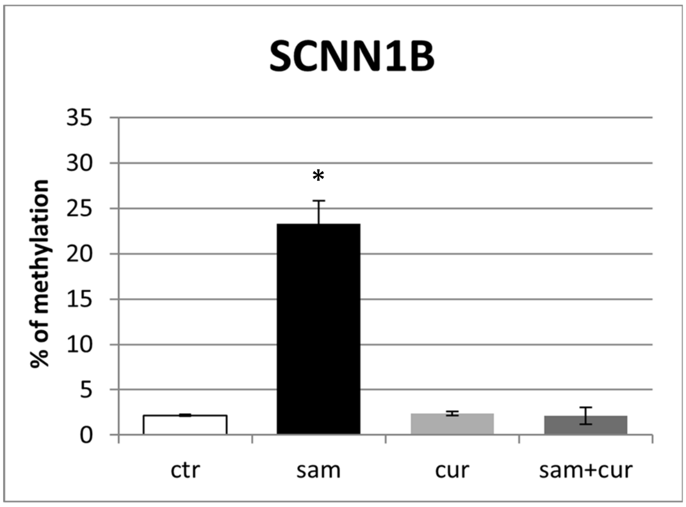

Figure S5 – Overall (CpG + non-CpG) DNA methylation of SCNN1B gene in CF primary bronchial epithelial cells, with the effect of SAM and curcumin on the pre-island and island zones taken together. Statistical significance of differences was evaluated by ANOVA followed by Bonferroni's post test: *P<0.001 (for sam *vs* untreated cells (ctr)). sam = S-adenosyl methionine, cur = curcumin.
